# BMC Sports Science, Medicine and Rehabilitation reviewer acknowledgement 2015

**DOI:** 10.1186/s13102-016-0030-4

**Published:** 2016-02-11

**Authors:** Clare Partridge

**Affiliations:** BioMed Central, Floor 6, 236 Gray’s Inn Road, London, WC1X 8HB UK

## Abstract

The editors of BMC Sports Science, Medicine and Rehabilitation would like to thank all our reviewers who have contributed to the journal in Volume 7 (2015).

**Silvia Achtzehn**

Germany

**Sufian S Ahmad**

Switzerland

**Cristine Alberton**

Brazil

**Jose Ramon Alvero Cruz**

Spain

**John Andrawis**

USA

**Francisco Areces Corcuera**

Spain

**Catharina Bäcklund**

Sweden

**David Barbado**

Spain

**Mary Barbe**

USA

**Patrick Barlow**

USA

**Ann Barr-Gillespie**

USA

**Jonathan Bartlett**

Australia

**Marcus Beasley**

UK

**David Berrigan**

USA

**Karen Birch**

UK

**Anthony Blazevich**

Australia

**Richard Bohannon**

USA

**Matteo Bonato**

Italy

**Elisabet Børsheim**

USA

**Maciej Buchowski**

USA

**Edward Burn**

UK

**Irene Cantarero-Villanueva**

Spain

**Fabrizio Caputo**

Brazil

**Romualdo Castillo-Lozano**

Spain

**Emil Chiauzzi**

USA

**Gary Chi Ching Chow**

Hong Kong

**Paohung Chung**

Taiwan

**Kenneth Clark**

USA

**Jamie Cooper**

USA

**James Croft**

Australia

**Jim Dickey**

Canada

**Rylee Dionigi**

Australia

**John Dixon**

UK

**James Dollman**

Australia

**Steven Elmer**

USA

**Yuri Feito**

USA

**Reed Ferber**

Canada

**Ivan Fernandez**

Spain

**Emerson Franchini**

Brazil

**Ellen Freiberger**

Germany

**Cas Fuchs**

Netherlands

**Laura-Anne Furlong**

UK

**Stuart Galloway**

UK

**Yang Gao**

Hong Kong

**Jeronimo Garcia-Romero**

Spain

**Jordan Glenn**

USA

**Kym Guelfi**

Australia

**Dustin Hardwick**

USA

**Liam Harper**

UK

**Emma Harris**

UK

**Stewart Head**

Australia

**Katja Heinicke**

Germany

**Trent Herda**

USA

**Jonathan Hoffman**

Belize

**Will Hopkins**

New Zealand

**Huanhuan Hu**

Japan

**Gerwyn Hughes**

UK

**Rocio Izquierdo**

Spain

**Kevin Jacobs**

USA

**Ana Jimenez-Cebrian**

Spain

**Mary Jung**

Canada

**Enda King**

Ireland

**Beat Knechtle**

Switzerland

**Yunsuk Koh**

USA

**Ljubica Konstantinovic**

Serbia

**G V Krishnaveni**

India

**Saravana Kumar**

Australia

**Kevin Kwong**

Hong Kong

**Ian Lahart**

UK

**Guang-Hua Lei**

China

**Christof Leicht**

UK

**Carl Lombard**

South Africa

**Mari Lundberg**

Sweden

**Hannu Luomajoki**

Switzerland

**Gemma Lyall**

UK

**Zuzana Machotka**

Australia

**Peter Malliaras**

Australia

**Theresa Mann**

South Africa

**Simon Marwood**

UK

**Myosotis Massidda**

Italy

**Álvaro Mateos-Angulo**

Spain

**Fermin Mayoral**

Spain

**Suzanne McDonough**

UK

**Shaun McLaren**

UK

**Steven McPhail**

Australia

**John Mercer**

USA

**Jose Antonio Merchán-Baeza**

Spain

**Dylan Morrissey**

UK

**Tanja Nauck**

Germany

**Kevin Netto**

Australia

**Sophia Nimphius**

Australia

**Jon Oliver**

UK

**Robert Otto**

USA

**Nick Owen**

UK

**David Oxborough**

UK

**Johnny Padulo**

Italy

**Giorgos Paradisis**

Greece

**Adriana Perez**

USA

**Marie-Josee Perrier**

Canada

**Stephanie S Pinto**

Brazil

**Fabiano Politti**

Brazil

**Mark Rakobowchuk**

UK

**Christoph Raschka**

Germany

**Chris Richter**

Ireland

**Chris Rissel**

Australia

**Jonatan Ruiz**

Spain

**Stephanie Schoeppe**

Australia

**Jonathon Senefeld**

USA

**Tzyy-Yuang Shiang**

Taiwan

**Ana Sousa**

Portugal

**Victoria Sprung**

UK

**Mike Stembridge**

UK

**Catherine Stewart**

UK

**Danny Taylor**

UK

**Apostolos Theodorou**

Greece

**Peter Tiidus**

Canada

**Moez Triki**

Tunisia

**Charilaos Tsolakis**

Greece

**James Turner**

UK

**Toyonobu Usuki**

Japan

**Tamara Valovich-Mcleod**

USA

**Raquel Vaquero-Cristóbal**

Spain

**Ana Velly**

Canada

**Elisa Vera-García**

Spain

**Cahouet Violaine**

France

**Ian Walshe**

UK

**Jodie Wilkie**

Australia

**Jonathan Williams**

UK

**Jim Winger**

USA

**Sara Winter**

Australia

**Junjie Xiao**

China

**Timothy Yam**

Hong Kong

**Ashril Yusof**

Malaysia

**Elias Zacharogiannis**

Greece

**Michael Zaki**

USA

**Johannes Zellner**

Germany

**Jian Zhang**

USA

